# Effects of vaginal microecology and immunity on the pregnancy outcome of cervical cerclage

**DOI:** 10.1186/s12905-022-01751-9

**Published:** 2022-05-14

**Authors:** Shengnan Cai, Yanting Wu, Li Zeng, Yiqian Ding

**Affiliations:** Affiliated Matern & Child Care Hospital of Nantong University, Nantong, 226000 Jiangsu China

**Keywords:** Cervical cerclage, Cervical incompetence, Vaginal microecology, IL-1β, IL-6, IL-8, TNF-α, Prognosis

## Abstract

**Objective:**

To investigate the effects of vaginal microecology and immune status on the pregnancy outcome of cervical cerclage.

**Methods:**

The clinical data of 125 patients with cervical incompetence who underwent transvaginal cervical cerclage in our hospital from January 2018 to January 2021 were collected, based on which the associations of vaginal microecology and related immune cytokines (IL-1β, IL-6, IL-8 and TNF-α) with the cervical cerclage outcome were explored.

**Results:**

All of the 125 patients had singleton pregnancies, who were aged 20–43 years, with a mean of (32.34 ± 5.17) years. The surgery was successful in 104 patients (full-term delivery or survival of premature infants), while unsuccessful in 21 patients (late miscarriage or death of premature infants), revealing a success rate of 83.20%. There were 70 full-term deliveries, 34 premature deliveries (28 survived while 6 died), and 15 late miscarriages. Univariate analysis revealed statistically significant differences in the timing of surgery, gestational age of cerclage, preoperative cervical canal length, genital tract infections and preoperative invasive procedures between the successful and unsuccessful groups (*P* < 0.05). Insignificant differences were found in the pre-pregnancy body mass index (BMI) (*P* > 0.05). According to the multivariate analysis results, cerclage timing and genital tract infections were independent risk factors for postoperative pregnancy failure (*P* < 0.05). The pathogen detection rates in the two groups of pregnant women were analyzed, finding significantly higher incidence of bacterial vaginosis (BV) in the unsuccessful group than in the successful group (*P* < 0.05). Inter-group comparison revealed that the positive rates for vaginal microenvironmental factors (LE, NAG, SNA, H_2_O_2_ and pH) were all significantly higher in the unsuccessful group than in the successful group (*P* < 0.05). Besides, the immune cytokine levels in the cervicovaginal secretions were also all significantly higher in the unsuccessful group than in the successful group (*P* < 0.05).

**Conclusion:**

The pregnancy outcome of patients undergoing cervical cerclage is associated with the imbalance of vaginal microecology and the levels of IL-1β, IL-6, IL-8 and TNF-α in cervicovaginal secretions.

Cervical incompetence, as one major cause of premature delivery and recurrent miscarriage resulting from painless cervical dilation in the second trimester, usually occurs at 18–22 weeks of pregnancy, with incidence between 0.1 and 1% [1]. Premature delivery is likely to increase the mortality and morbidity of the newborn, as well as the burden on the newborn families. Cervical incompetence occurs in 0.1–2.0% of premature delivery, which accounts for 15% of habitual miscarriage between 16 and 28 weeks of gestation [2]. The pathogenesis may be associated with such risk factors as congenital anatomical and histological abnormalities of cervix, multiple pregnancy, race, acquired cervical injuries (cervical conization, multiple cervical operations, delivery, etc.), polycystic ovary syndrome, as well as pelvic floor dysfunction. Transvaginal cervical cerclage, as a treatment for cervical incompetence, can reinforce the loose cervix, effectively prolong the pregnancy time, and improve the perinatal outcome [3]. However, there are many clinical factors that affect the success rate of the operation. Hence, through multiple factor analyses on the pregnant women undergoing transvaginal cervical cerclage in our hospital, this study explores the relevant influencing factors of the surgical success rate, with a view to improving the success rate of cervical cerclage in clinical settings.

## Materials and methods

### Information

#### General information

125 cases of cervical incompetence patients who underwent transvaginal cervical cerclage in the Maternal and Childcare Hospital of Nantong University from January 2018 to January 2021 were selected. All patients had singleton pregnancies, who were at 20–43 years of age, with a mean of (32.34 ± 5.17) years. This study has been approved by the Medical Ethics Committee of our hospital.

#### Inclusion and exclusion criteria

##### Inclusion criteria

(1) Pregestational diagnosis of cervical incompetence [4] (Meet one): ① History of one or more midterm miscarriages, premature delivery; previous damage of cervical tissues caused by cervical dilation, dystocia and other procedures. ② Detection of external cervix relaxation upon gynecological examination, where the No. 8 dilator can be inserted into the cervical canal without resistance; tubular enlargement at the confirmation of uterine isthmus confirmed by iodolography of the uterine cavity or tubes. (2) Gestational diagnosis of cervical incompetence [5]: ① Evident shortening of cervical canal with accompanying softening of the cervix, and even dilation of uterine orifice by > 2 cm; ② Width of internal cervical opening > 15 mm upon color Doppler ultrasound, showing wedge-shaped, funnel-shaped or amniotic sac protrusion.

##### Exclusion criteria

(1) Active uterine bleeding; (2) Fetal malformation or stillbirth; (3) Cervical incompetence caused by other factors (e.g. genetic, immune, endocrine, infectious, anatomic factors, etc.); (4) Twin pregnancy; (5) Severe gestational comorbidities, unsuitable for continuing gestation.

### Methods

#### Surgical procedure

The McDonald cerclage was adopted. After lumbar anesthesia, the vulva and vagina of the patients were disinfected in a bladder lithotomy position, and cervical traction was performed downward with Alex forceps. Trocar was inserted at the 11 o’clock position of the cervix and removed from the 10 o’clock position. Then, trocar was inserted at 8 o’clock and removed at 7 o’clock; inserted at 5 o’clock and removed at 4 o’clock; and subsequently inserted at 2 o’clock and removed at 1 o’clock. Knotting was made in the anterior fornix while avoiding the vascular plexus at the 3 and 9 o’clock positions, and 3 cm of thread was left for stitch removal. The internal cervical os should be indentified with a No. 4 Hegar dilator after suture. For those with amniotic sac protruding from the cervical canal, the amniotic sac was returned with water sac, and the knot should be closed to the cervical canal. Postoperatively, intravenous infusion of magnesium sulfate was given for fetus protection, uterine contraction inhibition and infection prevention. Besides, the patients were asked to rest in bed, abstain from sex, and receive B-ultrasound reexamination 2 weeks later. The stitches were removed before delivery or after 37 weeks of gestation. In the event of uninhibited contractions or signs of infection, the stitches should be removed at any time.

#### Criteria for surgical success and failure

The surgery was considered successful when the pregnancy outcome was full-term delivery or pregnancy < 28 weeks delivery but neonatal survival (successful group). Surgery failure was defined as abortion and neonatal death at < 28 weeks of gestation (unsuccessful group).

The information of the patients' age, gravidity and parity history, Gestational age of cerclage and length of cervical canal before pregnancy were collected. The pregnancy outcomes were observed after surgery, including Weeks of gestation, incidence of premature delivery, premature rupture of membranes, newborn birth weight and delivery method.

#### Timing of surgery

(1) Selective cervical cerclage: For 88 patients diagnosed with cervical incompetence prior to pregnancy, cerclage was performed at 14–18 weeks of pregnancy. (2) Therapeutic cervical cerclage: For 29 patients diagnosed with cervical incompetence by ultrasound during pregnancy, cerclage was performed at 16–28 weeks of pregnancy. (3) Emergency cervical surgery: For 8 patients whose fetal membranes reached or exceeded the external cervix upon gynecological examination, they were hospitalized for cervical cerclage immediately at 19–28 weeks of pregnancy.

#### Experimental methods

After vulvar disinfection, two sterile cotton swabs were used to collect the secretions from each pregnant woman at the 1/3 position of vaginal side wall and at the posterior vaginal fornix.

1. Five-indicator testing: Six biochemical indicators of vaginal secretions were tested using the kit manufactured by Likuo Development Company in Zhuhai, including the pH, leukocyte esterase (LE), hydrogen peroxide (H_2_O_2_), sialidase (SNA) and ß-N-acetylglucosaminidase (NAG).
2. Enzyme-linked immunosorbent assay (ELISA): Using the kit procured from R&D Systems in the USA, changes in the IL-1β, IL-6, IL-8 and TNF-α levels in patients' cervicovaginal secretions were detected with a ST-360 microplate reader (Kehua Bio-Engineering, Shanghai).

#### Observation indicators

(1) The pre-pregnancy BMI, gestational week of delivery, gestational age of intraoperative cerclage and cervical canal length during suture were recorded for all patients. The numbers of patients with preoperative genital tract infections and with preoperative invasive procedures (choriocentesis or amniocentesis) were counted. (2) The detection results of vaginal microenvironmental factors (LE, NAG, SNA, H_2_O_2_ and PH) were recorded. (3) The IL-1β, IL-6, IL-8 and TNF-α levels in cervicovaginal secretions were recorded. (4) The diagnostic method for bacterial vaginosis (BV): The cumulative score of *Lactobacillus*, *Mobiluncus* and *Gardnerella/Prevotella* scores under oil immersion microscope. ① *Gardnerella/Prevotella*: 0 point if the average count under oil immersion microscope was 0; 1 point if the count was 1; 2 points if the count was 2–5; 3 points if the count was 6–30; and 4 points if the count was > 30. ② *Mobiluncus*: 0 point if the average count under oil immersion microscope was 0; 1 point if the count was 1–5; and 2 points if the count was 6–30 or > 30. ③ *Lactobacillus*: 0 point if the average count under oil immersion microscope was > 30; 1 point if the count was 6–30; 2 points if the count was 2–5; 3 points if the count was 1; and 4 points if the count was 0. The cumulative score of the above 3 items was the Nugent score [6], which was normal if 0–3 points; critical if 4–6 points; and BV if ≥ 7 points.

### Statistical processing

SPSS 20.0 software was used for data processing. Measurement data were analyzed by t test and expressed as x ± s, while counting data were analyzed by χ^2^ test and expressed as percentages. Binary logistic regression was employed for multivariate analysis. Differences were considered statistically significant when *P* < 0.05.

## Results

Overall pregnancy outcome: 104 of 125 pregnant women had successful surgery, while 21 had unsuccessful surgery, showing a success rate of 83.20%. There were 70 full-term deliveries, 34 premature deliveries (28 survived while 6 died), and 15 late miscarriages.

Table 1 shows the univariate analysis of factors affecting pregnancy outcome: Statistically significant differences were found in the timing of surgery, gestational age of cerclage, preoperative cervical canal length, genital tract infections and preoperative invasive procedures between the successful and unsuccessful groups (*P* < 0.05). Comparison of pre-pregnancy BMI revealed insignificant inter-group difference (*P* > 0.05).

**Table 1:** Univariate analysis for pregnancy outcome after cervical cerclage

Factor	Outcome			
	Successful group (n = 104)	Unsuccessful group (n = 21)	χ^2^	*P*
Surgical timing			29.00	< 0.001
Selective cerclage	83	5		
Therapeutic cerclage	18	11		
Emergency cerclage	3	5		
Gestational age of cerclage			23.72	< 0.001
14–18 weeks	87	7		
> 18 weeks	17	14		
Cervical length (mm)			18.63	< 0.001
≥ 2.5 cm	86	8		
< 2.5 cm	18	3		
Preoperative invasive procedures			10.88	0.004
Yes	8	7		
No	96	14		
Genital tract infections			12.37	< 0.001
Yes	33	14		
No	71	7		
BMI (kg/m^2^)			0.85	0.474
≥ 25.5	48	12		
< 25.5	56	9		

Table 2 shows the multivariate analysis of factors affecting pregnancy outcome: Pregnancy outcome was taken as the dependent variable (successful = 0, unsuccessful = 1), while factors differing significantly in the univariate analysis (surgical timing, gestational age of cerclage, cervical canal length and preoperative invasive procedures) were used as the independent variables. Value assignment was performed, and the surgical timing (selective cerclage = 1, therapeutic cerclage = 2, emergency cerclage = 3), gestational age of cerclage (14–18 weeks = 1, > 18 weeks = 2), cervical canal length (≥ 2.5 cm = 1, < 2.5 cm = 2), genital tract infections (no = 1, yes = 2), and preoperative invasive procedures (no = 1, yes = 2) were incorporated into the logistic regression model. The results showed that the timing of cerclage and the genital tract infections were independent risk factors for postoperative pregnancy failure (*P* < 0.05).

**Table 2:** Multivariate analysis for pregnancy outcome after cervical cerclage

Factor	Outcome				
	β	S.E	Wald	OR (95% CI)	*P*
Surgical timing	− 3.230	0.960	11.322	0.040 (0.006–0.260)	0.001
Gestational age of cerclage	2.374	1.425	2.776	10.739 (0.658–175.289)	0.096
Cervical canal length	− 2.048	1.077	3.619	0.129 (0.016–1.064)	0.057
Preoperative invasive procedures	1.538	0.862	3.182	4.655 (0.859–25.224)	0.074
Genital tract infections	2.145	0.809	7.037	8.546 (1.751–41.703)	0.008
Pre-pregnancy BMI	0.447	0.737	0.367	1.563 (0.369–6.629)	0.544

Table 3 shows the inter-group comparison of BV incidence: The pregnant women in the unsuccessful group exhibited significantly higher incidence of BV than those in the successful group (*P* < 0.05).

**Table 3:** Effects of BV on the pregnancy outcome after cervical cerclage

Nugent Nugent scores			
Group	0–3	4–6	≥ 7
Successful group (n = 104)	101 (97.11)	3 (2.89)	0 (0)
Unsuccessful group (n = 21)	2 (9.52)	17 (80.96)	2 (9.52)
χ^2^	92.43	79.23	10.07
*P*	< 0.001	< 0.001	0.027

Table 4 shows the positive rates for all vaginal microenvironmental factors were significantly higher in the unsuccessful group than in the successful group (*P* < 0.05).

**Table 4:** Effects of vaginal microenvironmental factors on pregnancy outcome after cervical cerclage

Group	Factor				
	LE	NAG	SNA	H2O2	PH
Successful group (n = 104)	24	4	4	2	4
Unsuccessful group (n = 21)	12	5	8	11	13
χ^2^	9.888	10.421	23.616	48.838	50.121
*P*	0.003	0.007	< 0.001	< 0.001	< 0.001

Table 5 shows all the immune cytokine levels in the cervicovaginal secretions were significantly higher in the unsuccessful group than in the successful group (*P* < 0.05).

**Table 5:** Effects of vaginal immune status on pregnancy outcome after cervical cerclage

Group	Factor			
	IL-1β	IL-6	IL-8	TNF-α
Successful group (n = 104)	103.80 ± 13.60	12.87 ± 2.92	1653.09 ± 77.07	5.76 ± 0.40
Unsuccessful group (n = 21)	1012.38 ± 32.96	15.28 ± 2.60	2616.82 ± 134.97	11.25 ± 1.51
T	− 124.199	− 3.504	31.695	16.552
*P*	< 0.001	0.001	< 0.001	< 0.001

## Discussion

### Research background

Cervical incompetence is also known as the cervical laxity or the internal os insufficiency. In recent years, a growing number of women have developed cervical incompetence in China. If no active, effective treatment is given or in the case of improper treatment, the pregnant women will have increased risk of adverse events such as miscarriage and premature delivery, probably accompanied by severer consequences. Research has shown that the premature birth rate for women with cervical incompetence is 3.3 times that of those without cervical incompetence.

### Correlation between vaginal microecology and cerclage outcome

Transvaginal cervical cerclage, as an effective surgical procedure for preventing miscarriage and premature delivery, has been used for more than 60 years, which features short operation time, fast postoperative recovery and easy acceptance by patients [7]. This procedure can avoid cervical os dilation and lower uterine extension, and share weights of fetus and amniotic fluid with the cervix, thereby effectively prolonging the gestational age of cervical incompetence patients and ameliorating their pregnancy outcome [8]. In this study, 104 of 125 pregnant women had successful surgery, while 21 had unsuccessful surgery, with a success rate of 83.20%, suggesting higher success rates of transvaginal cervical cerclage. In this study, the gestational age and the weeks of delayed pregnancy were both higher in the successful group than in the unsuccessful group, suggesting that the transvaginal cervical cerclage can prolong the gestational age of patients. According to univariate analysis, the surgical timing, gestational age of cerclage, preoperative cervical canal length, genital tract infections and preoperative invasive procedures were the risk factors for cervical cerclage failure (*P* < 0.05). Multivariate analysis revealed that the cerclage timing and genital tract infections were independent risk factors for postoperative pregnancy failure (*P* < 0.05).

Diverse microorganisms reside in the vaginal mucosa of women, which restrict and interact with each other to maintain the healthy vaginal microenvironment. The imbalance of vaginal microecology is governed by the etiological, environmental and host factors. Pregnancy is a special physiological period, during which the physiological hormonal variations easily cause changes in the vaginal flora environment, thereby resulting in imbalance of vaginal microecology [9, 10]. This greatly increases the risk of various types of vaginal inflammation. However, extensive research evidence demonstrates that the genital tract infections caused by vaginal microecological imbalance are an important cause of premature delivery. Through analysis of vaginal flora density between the successful and unsuccessful groups, this study finds higher density of vaginal flora among the pregnant women in the unsuccessful group than those with full-term pregnancy. This suggests probable association of the cervical cerclage failure with the vaginal microecological changes.

Research has shown that *Lactobacillus* is the most common normal flora in the vagina, which is a resident vaginal flora in normal mature women. Its function is to lower the vaginal pH by converting glycogen into lactic acid, so that the vaginal pH of normal healthy women can be maintained between 3.8 and 4.2, which is acidic and help sustain the vaginal flora balance [11]. According to the results of this study, the pregnant women in the unsuccessful group had markedly higher pH than those in the successful group. The concentration of H_2_O_2_ has been shown to be associated with the number of lactobacilli. If the H_2_O_2_ test result is negative, it indicates a high vaginal level of lactobacilli and a good flora state. As suggested by the results of this study, the unsuccessful group exhibited considerably higher positive rate of H_2_O_2_ than the successful group (*P* < 0.05). LE (+) indicates damage of local vaginal mucosa [12]. NAG can assess the severity of vaginal damage, which is involved in both the invasion and metastasis of vaginal inflammation [13]. An elevated level of SNA, which is secreted by Gardnerella vaginalis and anaerobic bacteria, represents an increase in the Gardnerella vaginalis secretion. The results of the present study showed that the positive rates for LE (+), NAG (+), SNA (+), pH > 4.5 and H_2_O_2_ in the unsuccessful group were significantly higher than those in the successful group (*P* < 0.05). This suggests that the vaginal microecology is an important factor affecting the pregnancy outcome after cervical cerclage, which provides new ideas for the prevention and management of miscarriage and premature delivery.

### Correlations of IL-1β, IL-6, IL-8 and TNF-α levels in vaginal secretions with cerclage outcome

IL-1, as one of the foremost regulators of inflammatory response, affects almost all types of cells [14]. IL-1β is an important mediator of inflammatory response and a major proinflammatory cytokine. In a study by Barlik et al. [15], elevated IL-1β concentration in cervical secretions was linked to increased risk of premature delivery, showing agreement with the finding of the present study. IL-6 is a pluripotent inflammatory cytokine, whose level in amniotic fluid increases upon occurrence of intraamniotic infection. During inflammatory response, IL-6, as a cytokine secreted following infection and stimulation, can promote the maturation and differentiation of T and B lymphocytes, mediate the acute inflammatory response, and enhance the chemokine effects. Research has shown that elevated serum IL-6 was closely associated with such adverse obstetric events as premature rupture of membranes, intrauterine infections and premature delivery, proposing the potential of serum IL-6 as a predictor for early intrauterine infection and threatened premature delivery [16]. In this study, the IL-6 concentration in vaginal secretions of women having unsuccessful cervical cerclage was higher than those having successful cerclage, which further verified the involvement of IL-6 in the occurrence of premature delivery. IL-8 can promote the chemotaxis of neutrophils and lead to cervical ripening, which is closely associated with premature delivery. Spontaneous premature delivery has been reported to be correlated with elevated IL-8 in cervical mucus and short cervix [17, 18]. This also agrees with the finding of the present study. TNF-α, a pluripotent cytokine, is a product of maternal and fetal tissues. By increasing the prostaglandin production, it activates the myometrium, induces the activation and apoptosis of matrix metalloproteinase, and promotes the occurrence of premature delivery, thereby affecting the pregnancy outcome [19]. Although serum TNF-α level is low in physiological pregnancy, it may rise sharply upon infections, especially in the case of genitourinary system infections. Sidelnikova and Sukhikh found that the serum TNF-α level of pregnant women who were at risk of premature delivery in the third trimester was ~ 9 times higher than that of normal pregnant women [20]. In this study, the TNF-α level in cervicovaginal secretions of women having unsuccessful cerclage was higher than those having successful cerclage. This result agrees with that of serum TNF-α level, which further proves the involvement of TNF-α in the occurrence of premature delivery.

BV is a common bacterial infection of vagina in clinical settings. As a mixed infection caused predominantly by *Gardnerella bacillus*, BV can cause vaginal microecological disorders, leading to reduction or even loss of *Lactobacilli* and excessive reproduction of pathogenic bacteria, thereby further increasing the incidence of threatened premature delivery in pregnant women. This study found considerably higher failure rate of cervical cerclage in patients with BV than those in the BV-negative group. The IL-1β, IL-6, IL-8 and TNF-α levels in vaginal secretions of the enrolled pregnant women were examined, revealing significantly higher expressions of inflammatory cytokines in the unsuccessful group than in the successful group (*P* < 0.05). This is probably associated with the stimulation of IL-1β, IL-6, IL-8 and TNF-α productions by Gardnerella vaginalis in vitro through choroidal deciduas [17, 21].

### Conclusion

The pregnancy outcome of patients undergoing cervical cerclage is closely linked to the imbalance of vaginal microecology. Elevated levels of IL-1β, IL-6, IL-8 and TNF-α in cervicovaginal secretions are probably associated with the decreased clearance of pathogenic bacteria and the enhancement of inflammatory response caused by vaginal microecological imbalance. Thus, in pregnant women, the imbalance of vaginal microenvironment increases the risk of premature delivery, which has adverse effects on both the pregnant women and fetuses. Assessment of microecological changes can better guide the clinical treatment, increase the success rate of cervical cerclage, and improve the maternal and infant outcomes.

## Data Availability

All data generated or analysed during this study are included in this published article.
